# Supplementary material for: Cryo-EM structure of the complete E. coli DNA gyrase nucleoprotein complex
Source: Nat Commun. 2019 Oct 30;10:4935. doi: 10.1038/s41467-019-12914-y (PMC6821735; doi:10.1038/s41467-019-12914-y)
Supplement: Supplementary file 1 — Supplementary Information [file 41467_2019_12914_MOESM1_ESM.pdf]

Supplementary information

Cryo-EM structure of the complete *E. coli* DNA gyrase nucleoprotein complex

Vanden Broeck *et al.*

TABLE OF CONTENT

Supplementary Figure 1. Purification and negative supercoiling activity of reconstituted *E. coli* DNA gyrase

Supplementary Figure 2. Cryo-EM data acquisition and *ab-initio* model generation

Supplementary Figure 3. Flow chart of the cryo-EM data processing

Supplementary Figure 4. Cryo-EM statistics of the overall DNA-bound *E. coli* DNA gyrase and DNA-binding/cleavage domain in closed and pre-opening states

Supplementary Figure 5. High and intermediate resolution cryo-EM maps of the different functional domains fitted into the low resolution cryo-EM map of the overall complex

Supplementary Figure 6. DNA gyrase overall geometry analysis

Supplementary Figure 7. Comparison of the X-ray and cryo-EM structures of *E. coli* DNA gyrase at the GyrB-GyrA extremities interface

Supplementary Figure 8. Conservation of the GHKL/Transducer domains and R286 residue in bacteria

Supplementary Figure 9. Thermal stability analysis and DNA stimulated ATP hydrolysis activity

Supplementary Figure 10. Quaternary and tertiary changes associated with G-segment binding and opening after cleavage

Supplementary Figure 11. Metal-binding site associated with cleavage of DNA by DNA gyrase

Supplementary Figure 12. CTD β-pinwheel acidic tail modeling

Supplementary Figure 13. Comparison of the *E. coli* and *S. aureus* NBTI binding site

Supplementary Figure 14. Experimental map quality around the NBTI molecule

Supplementary Table 1. Asymmetric oligonucleotides sequences

Supplementary Table 2. Data collection, processing and refinement statistics

Supplementary Table 3. Missing DNA gyrase sequence elements modeled in this study

Supplementary Table 4. Primer sequences used for the plasmids mutagenesis

Supplementary References

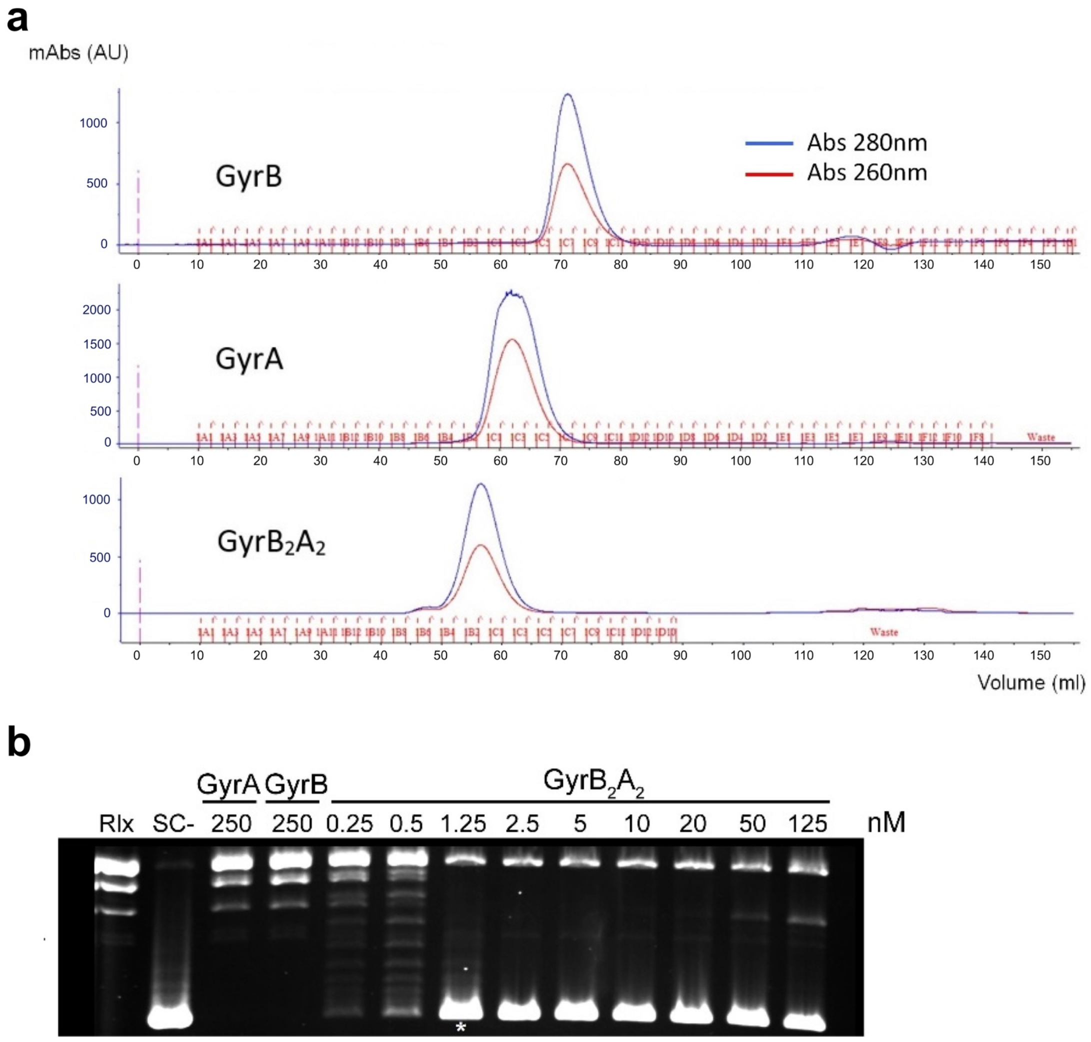

Supplementary Figure 1. Purification and negative supercoiling activity of reconstituted *E. coli* DNA gyrase. a. Comparison of the elution volumes of the GyrB subunit, GyrA subunit and the reconstituted $GyrA_2B_2$ DNA gyrase showing a clear shift on a gel filtration column (Superdex S200 16/60). **b.** Negative supercoiling activity of the reconstituted *E. coli* DNA gyrase. Protein concentrations are listed in nM holoenzyme and asterisk indicates concentration of enzyme needed to supercoil the substrate in 30 min. Negative and positive controls are shown as relaxed (Rlx) or negatively supercoiled DNA species (SC-), respectively. The source data are provided as a Source Data file.

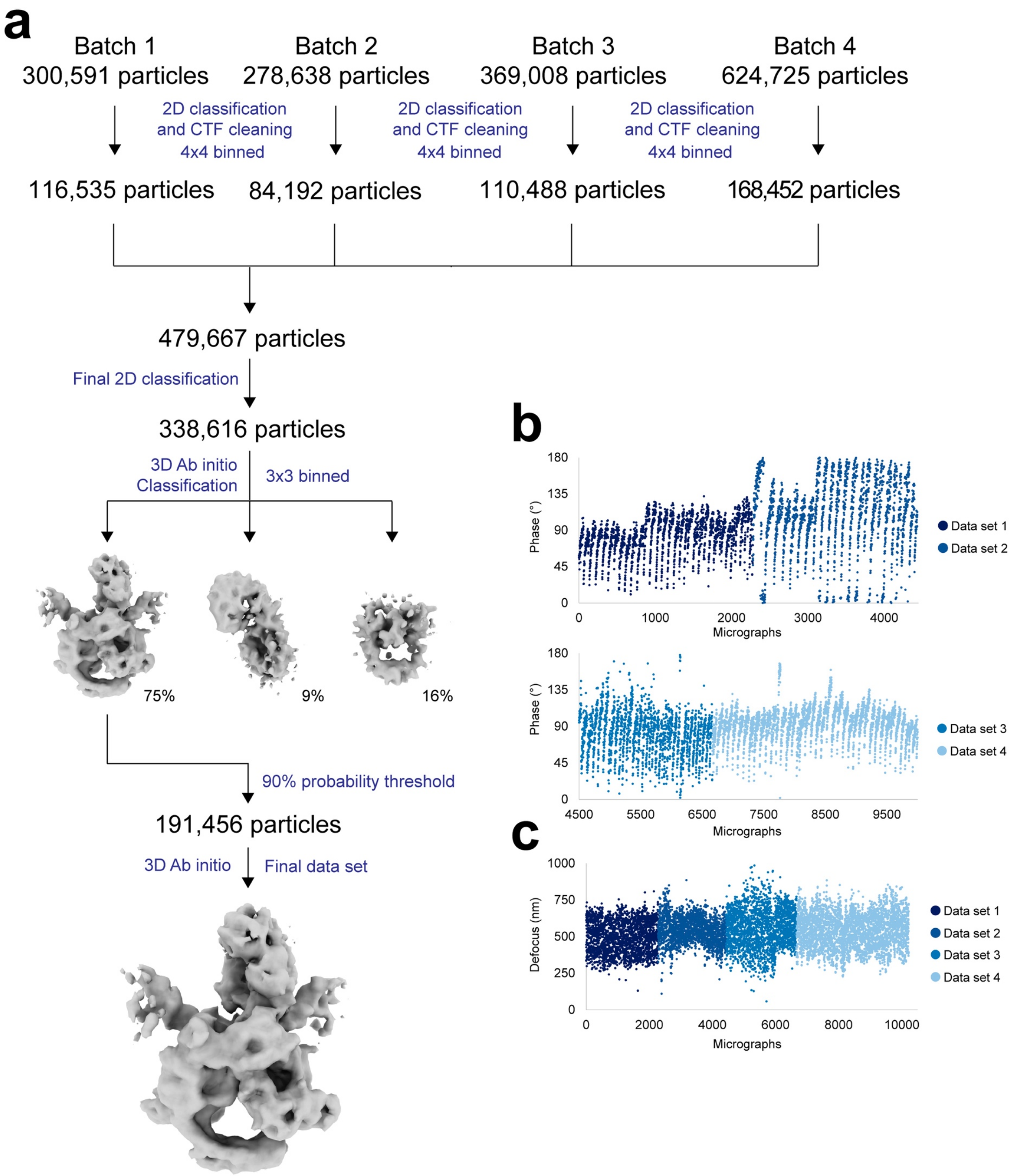

Supplementary Figure 2. Cryo-EM data acquisition and *ab-initio* model generation. a. Flow chart of data processing from 2D classification to *ab-initio* model generation. The particle numbers are indicated at each step. **b.** Volta phase plate phase shift throughout the dataset. **c.** Defocus estimation history throughout the dataset.

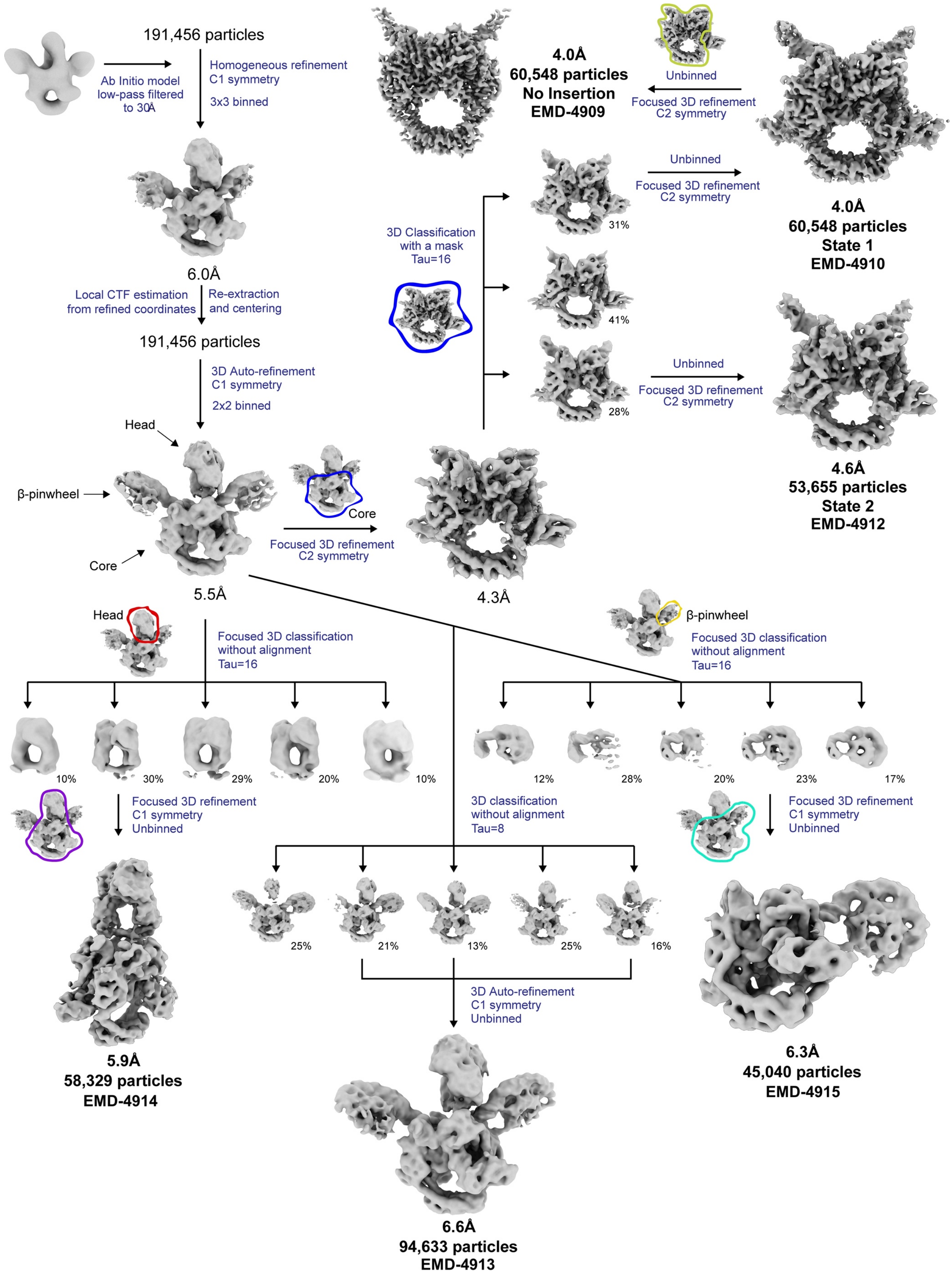

Supplementary Figure 3. Flow chart of the cryo-EM data processing. *Ab-initio* model was refined in cryoSPARC [1] to 6.0 Å overall using 191,456 particles. Using the refined coordinates, particles were re-extracted and centered and per-particle CTF estimation was performed with GCTF [2]. Using this new particles stack, the previous structure was refined to 5.5 Å overall. Using

RELION2 [3,4], focused 3D classification with and without alignment followed by focused 3D refinement allowed to solve 6 new structures: the DNA-binding/cleavage domain in closed state (with and without TOPRIM insertion) at 4.0 Å and pre-opening state at 4.6 Å, the DNA-binding/cleavage domain with the ATPase domain at 5.9 Å, the DNA-binding/cleavage domain with the β-pinwheel 6.3 Å and the overall complex with improved quality of the flexible regions at 6.6 Å. The particle numbers for the final refinement are indicated. 3x3, 2x2 binned and unbinned structures corresponds to pixel sizes of 2.64, 1.76 and 0.88 Å/px, respectively.

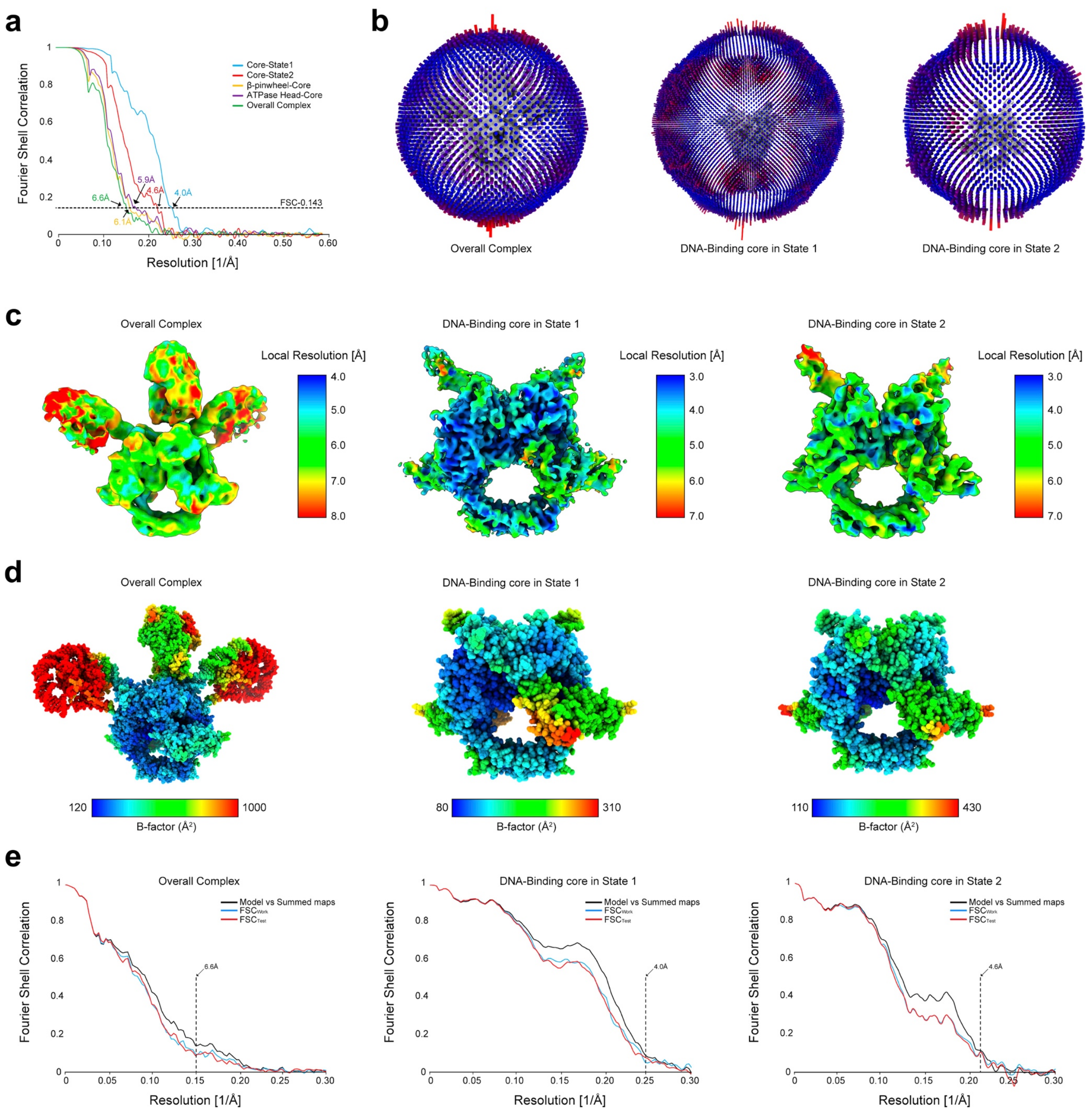

Supplementary Figure 4. Cryo-EM statistics of the overall DNA-bound *E. coli* DNA gyrase and DNA-binding/cleavage domain in closed and pre-opening states. a. FSC plots and resolution estimation using the gold-standard 0.143 criterion generated from RELION2 [3,4]. **b.** Angular distribution plots generated from RELION2. **c.** Final refined map colored according to local resolution calculated with Blocres [5]. **d.** Atomic models refined in the corresponding cryo-EM maps colored according to the B-factors. **e.** Cross-validation FSC curves for the corresponding refined models versus unfiltered half maps (the one used in the refinement, FSC_{work}, and the other half, FSC_{free}) and the unfiltered summed maps.

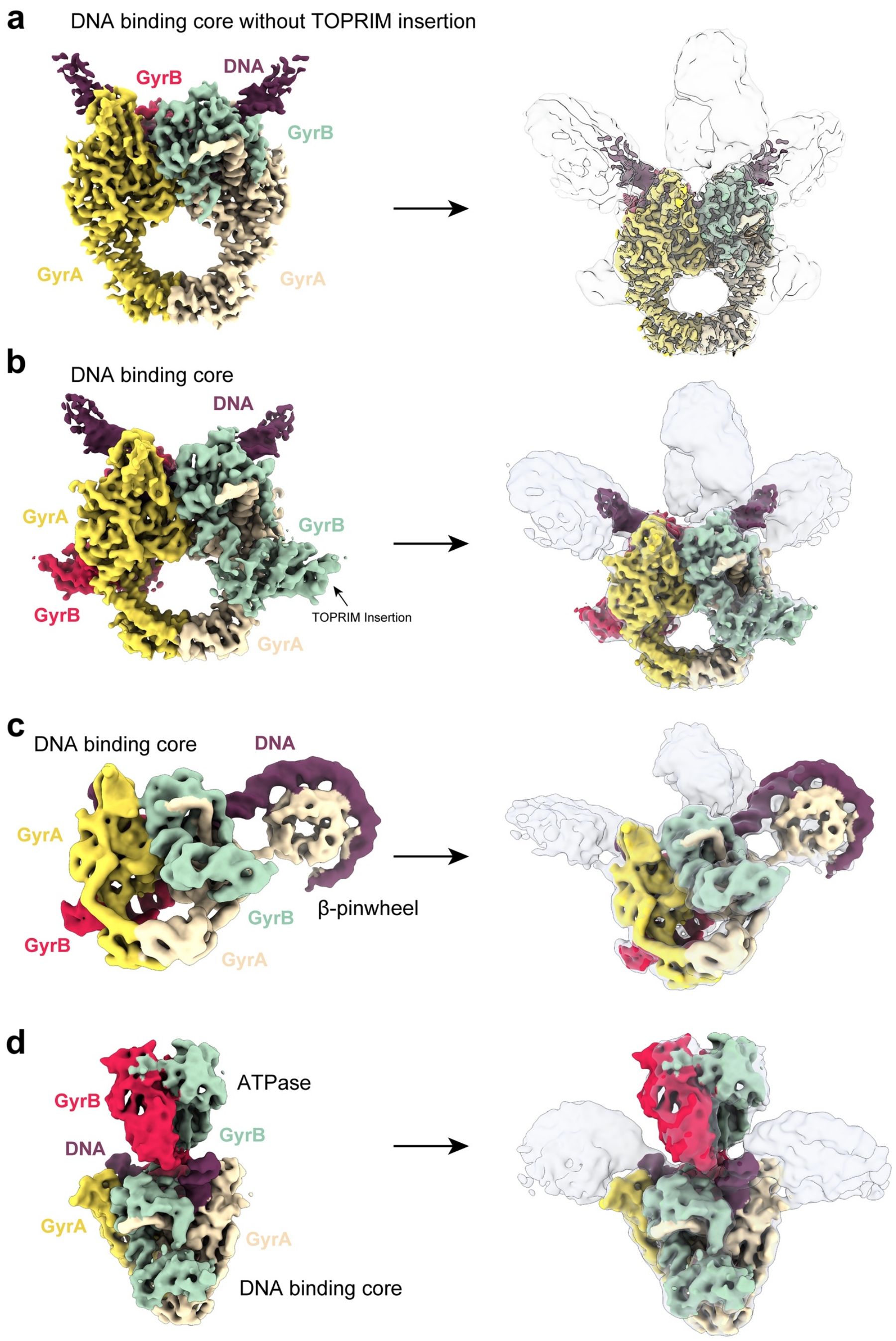

Supplementary Figure 5. High and intermediate resolution cryo-EM maps of the different functional domains fitted into the low resolution cryo-EM map of the overall complex. a. The DNA-binding/cleavage domain in closed state lacking the TOPRIM insertion solved at 4.0 Å resolution fitted in the 6.6 Å map of the overall complex. **b.** The DNA-binding/cleavage domain in closed state solved at 4.0 Å resolution fitted in the 6.6 Å map of the overall complex. **c.** The DNA-binding/cleavage domain in closed state with the β-pinwheel domain solved at 6.3 Å resolution fitted in the 6.6 Å map of the overall complex. **d.** The DNA-binding/cleavage domain in closed state with the ATPase domain solved at 5.9 Å resolution fitted in the 6.6 Å map of the overall complex. The different maps were used to build and refine the atomic model of the entire complex.

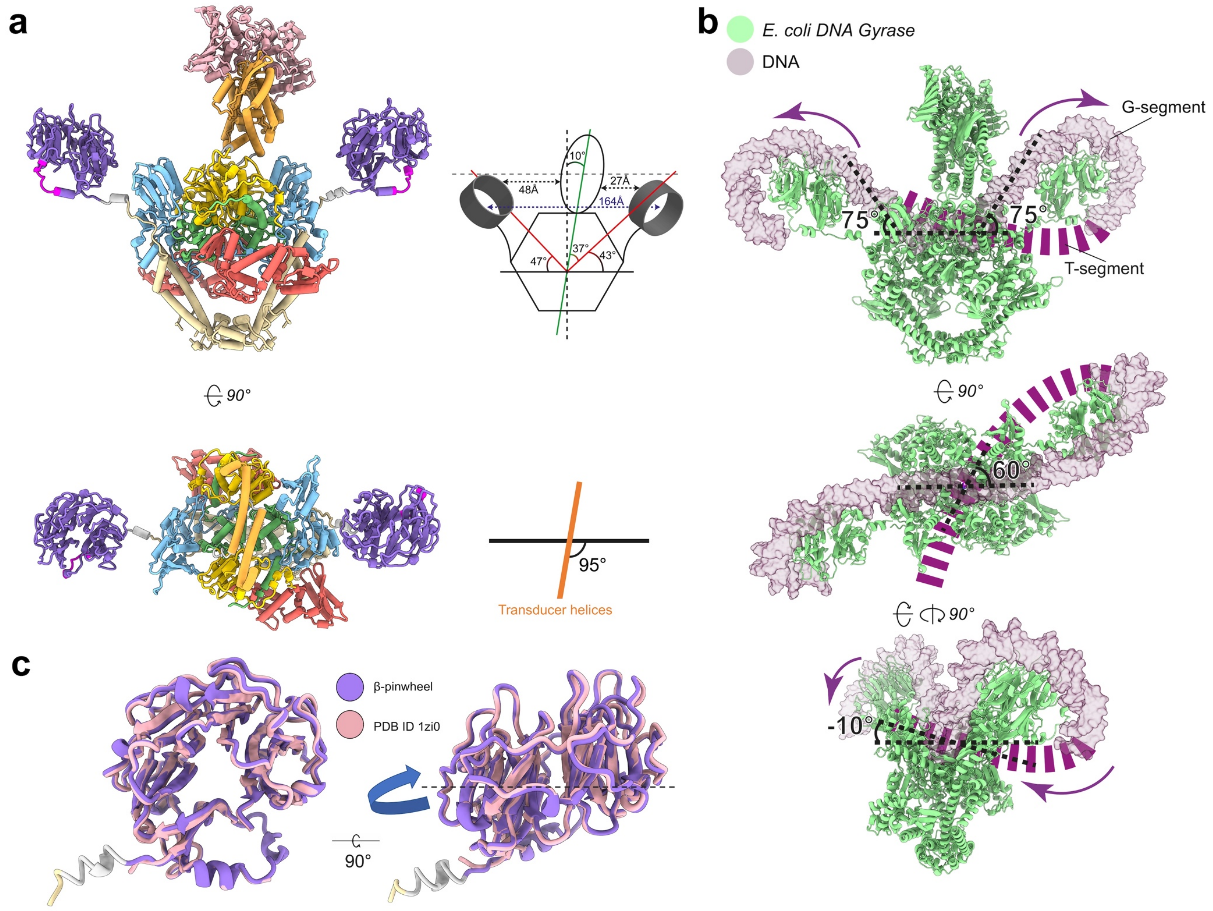

Supplementary Figure 6. DNA gyrase overall geometry analysis. a. Upper panel: a schematic representation of the domains' orientation is shown for the gyrase complex model. The DNA-binding/cleavage domain (DNA- and C-gate) is depicted as a hexagon, the GyrB ATPase domain (N-gate) as an ellipse, and the CTD β-pinwheel domains as disks. Red and green solid lines indicate the β-pinwheel and ATPase domain planes, respectively. The ATPase domain bends toward one β-pinwheel with an angle of ~10°. This positions the ATPase domain at a distance of 26 Å from the β-pinwheel. The second β-pinwheel is 48 Å from ATPase domain. The β-pinwheels are located in an upper position and are distributed asymmetrically on each side of the DNA gate (47° versus 43°). Lower panel: Top view of the structure with the upper part of the ATPase domain omitted. The ATPase domain transducer α-helices in magenta and forms a ~95° angle with the DNA-binding/cleavage domain. The color code is the same as in Fig. 1a. DNA is omitted for clarity. **b.** Orthogonal views of the *E. coli* DNA gyrase structure complex. In the top and side views, the ATPase domain has been omitted for clarity. The spatial arrangement of the β-pinwheels induces an overall ~150° bending of the DNA. The 130 bp DNA duplex (purple) is chirally wrapped around the β-pinwheel in an orientation that is consistent with the formation of a 60° angle positive crossover when the T-segment path is extrapolated in the DNA-gate groove formed by the TOPRIM-WHD and TOWER domains. The angle of the T-segment entering the gyrase core is of -10° respective to the plan of the DNA-binding groove. **c.** Superimposition of the β-pinwheel structure from the *E.coli* nucleoprotein complex to the crystal structure of the isolated domain published by Ruthenburg et *al.*[10]

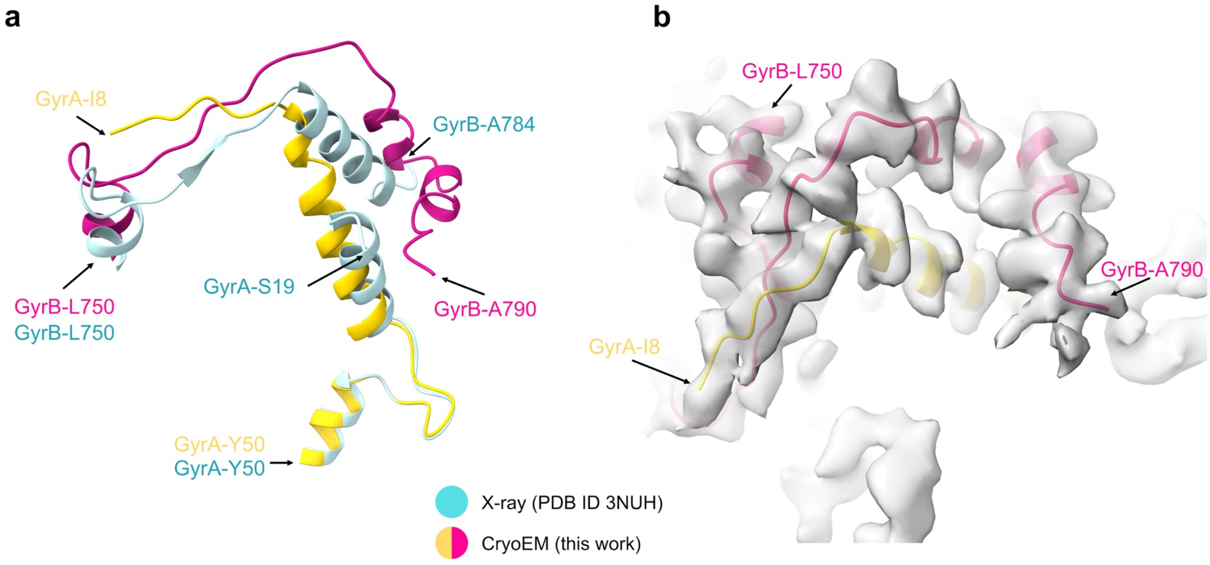

Supplementary Figure 7. Comparison of the X-ray and cryo-EM structures of *E. coli* DNA gyrase at the GyrB-GyrA extremities interface. a. After several rounds of manual building and refinement, the resulting cryo-EM atomic model was superimposed on the X-ray structure (PDB ID 3NUH) [6] in the area of the GyrB C-terminal (L750-A790) and GyrA N-terminal (I8-Y50) extremities. The superimposition of the structures shows a misalignment of both GyrB and GyrA extremities. **b.** The quality of the cryo-EM map allowed to unambiguously build and correct this area.

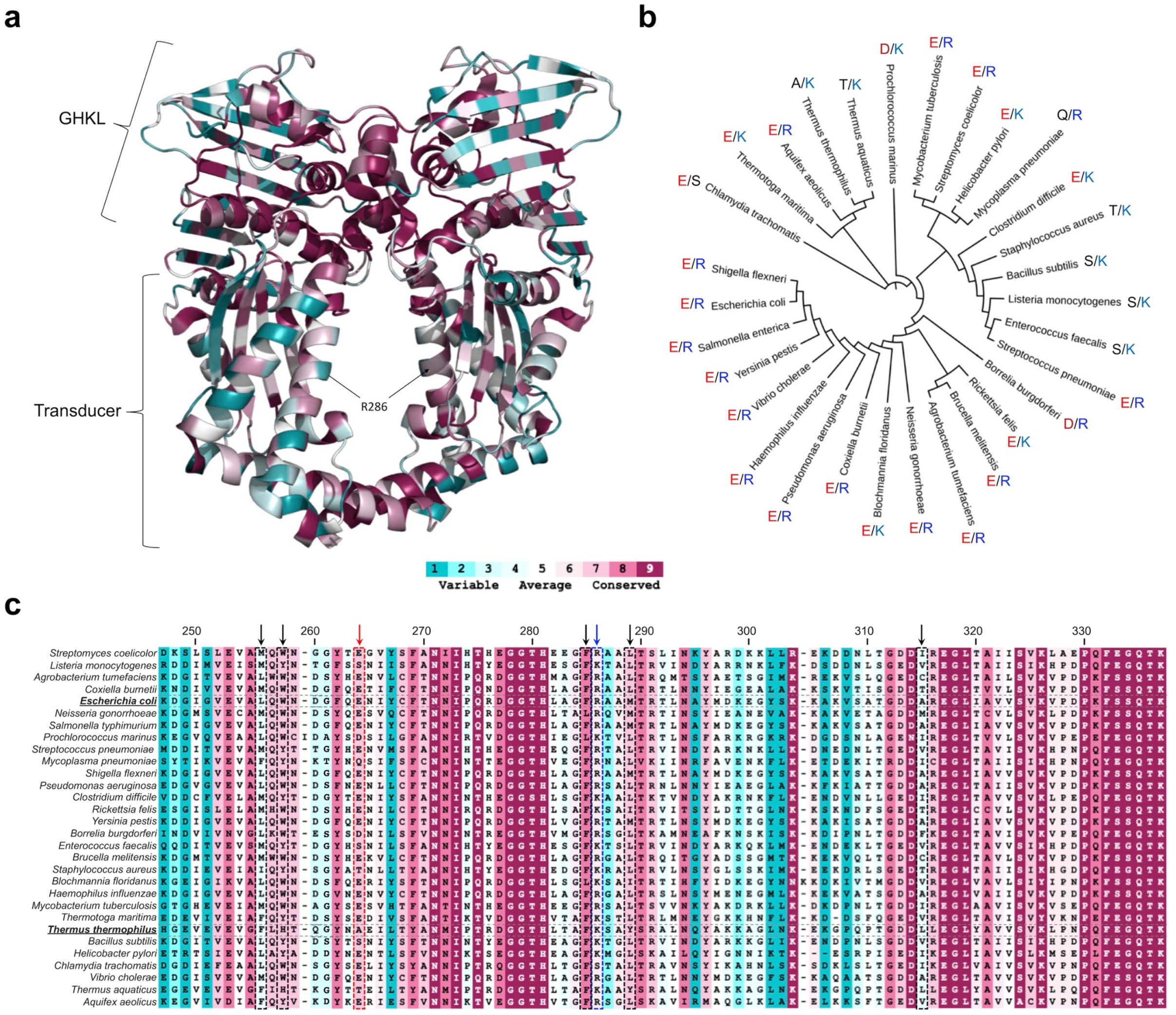

Supplementary Figure 8. Conservation of the GHKL/Transducer domains and R286 residue in bacteria. a. Atomic model of the ATPase and transducer domains (PDB ID 1EI1) [7] colored by conservation level for each residue among 30 species within Gram-positive and Gram-negative bacteria. All the residues interacting with ATP in the GHKL domain are highly conserved while residues in the transducer show a smaller degree of conservation. **b.** Phylogenetic tree generated using a multiple alignment of the ATPase/transducer sequences from 30 bacterial species (listed in c). The R286-E264 couple (numbering based on *E. coli* sequence) is indicated for each species. R286 is found either as an arginine, mostly in Gram negative bacteria, or as a lysine in Gram positive bacteria. **c.** Multiple alignment of the ATPase/Transducer domain focused on the transducer domain. Black arrows indicate the residues implicated in hydrophobic contacts stabilizing the transducer beta-strands and alpha helices. The blue arrow indicates the R286 and homologous residues within the 30 species. The red arrow indicates the E264 and homologous residues within the 30 species.

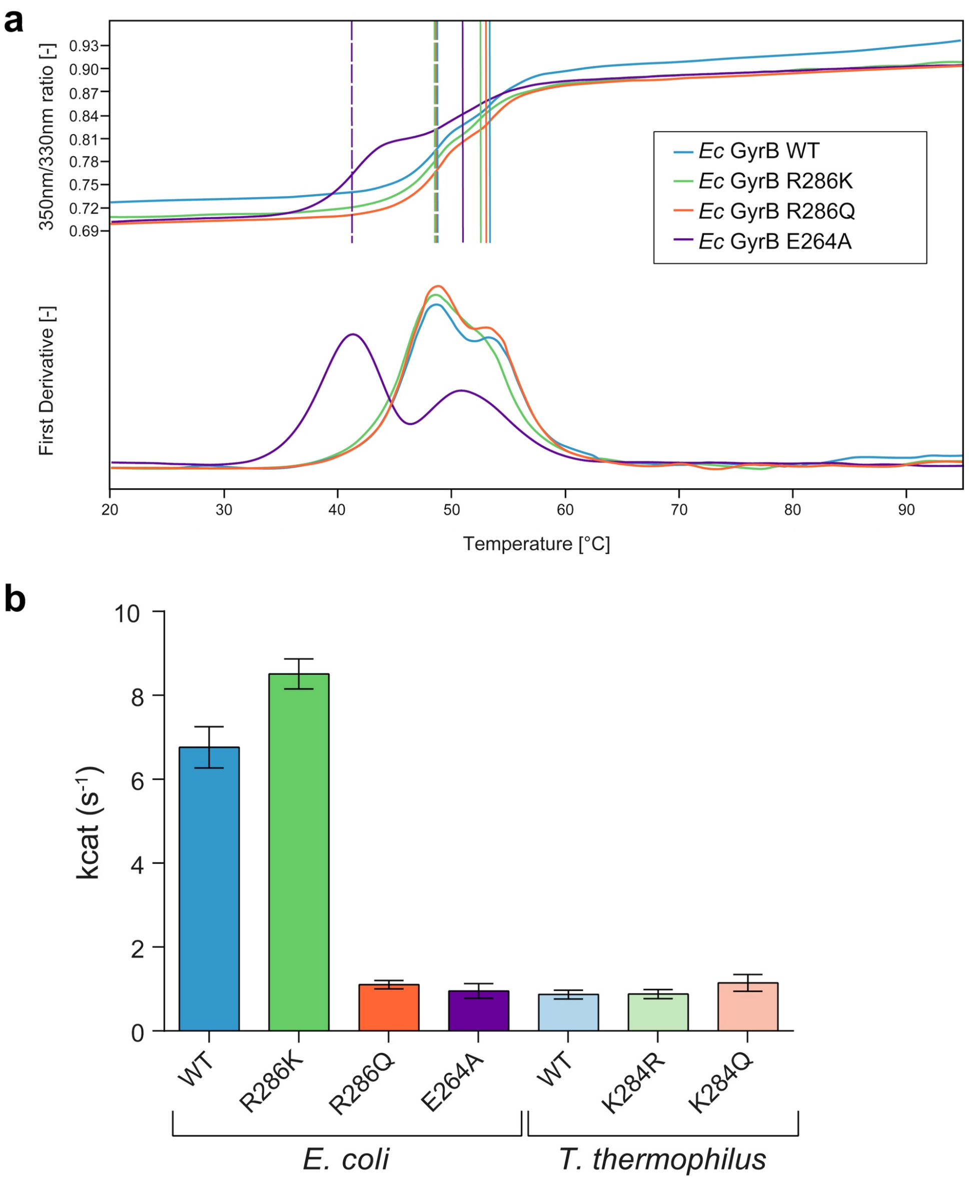

Supplementary Figure 9. Thermal stability analysis and DNA stimulated ATP hydrolysis activity. **a**. The thermal stability of the *E. coli* WT, R286K, R286Q and E264A GyrB domains was measured by differential scanning fluorimetry based on the intrinsic fluorescence of tryptophan. The denaturation curve of the WT GyrB and of the R286K, R286Q mutants shows no major difference. In contrast, the E264A mutant thermal denaturation curve is shifted towards lower temperatures (upper panel). The first derivative (lower panel) displays two peaks consistent with a multidomain protein with no significant shift of the main temperature transition peak except for the E264A mutant which displays a 8°C lower transition, compared to the WT. **b.** DNA stimulated ATP hydrolysis activity of WT, R286K, R286Q and E264A *E. coli* DNA gyrase and WT, K284R and K284Q *T. thermophilus* DNA gyrase. The mutation R286K shows no negative effect on the ATPase activity of the *E. coli* DNA gyrase. The R286Q and E264A show a 6-fold and 8-fold reduction of their ATPase activities, respectively. The K284R and K284Q mutations have no effect on ATPase activity of *T. thermophilus* DNA gyrase. Errors bars represent the standard deviation (SD) for measurements recorded in triplicate for each sample. The source data are provided as a Source Data file.

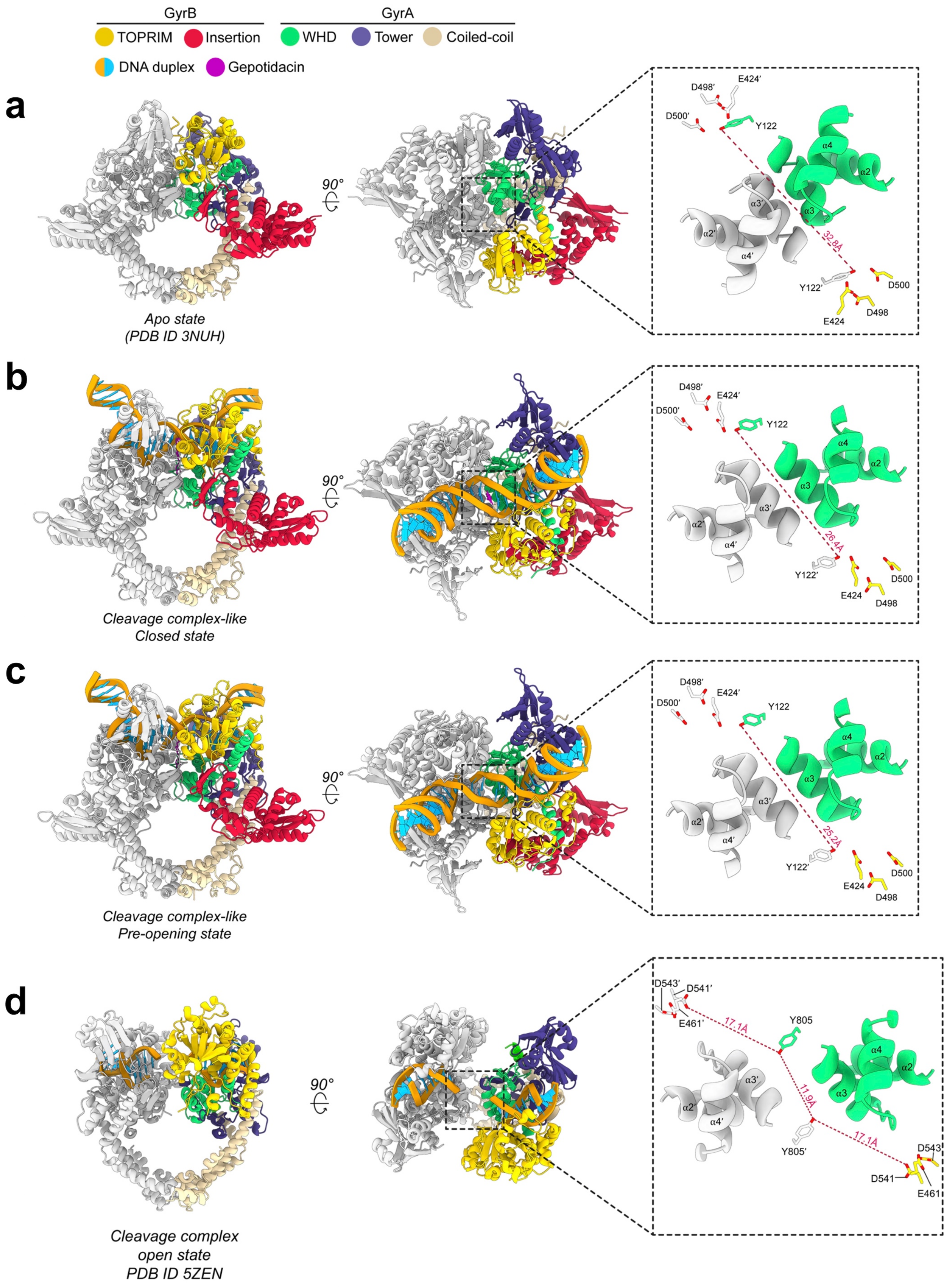

Supplementary Figure 10. Quaternary and tertiary changes associated with G-segment binding and opening after cleavage. Orthogonal views of the *E. coli* DNA-binding/cleavage domain in different states. The inset shows selected structural elements (alpha helices α2, α3 and α4) lining the bottom side of the G-segment groove and key catalytic groups in the *apo* [6] (A) closed

cleavage complex (B) and pre-opening cleavage complex (C) and open cleavage complex [8] (D) conformations. For each conformation, the catalytic tyrosines, the Mg^{2+}-binding residues, and the distance between the two catalytic tyrosines are shown to illustrate structural changes in the DNA-gate during the Apo-to-closed and closed-to-pre-opening transitions. Residues from different homodimer (GyrB-GyrA or hsTop2β) are colored differently and marked by a quotation mark.

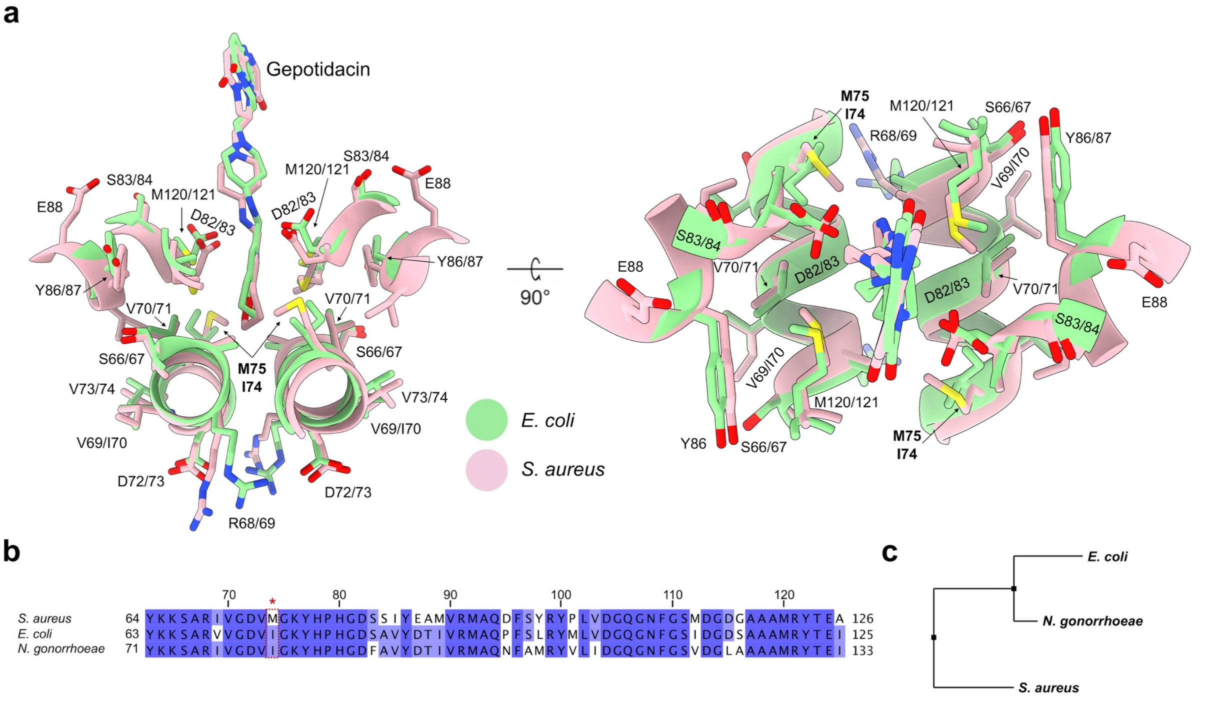

Supplementary Figure 11. Comparison of the *E. coli* and *S. aureus* NBTI binding site. a. Superimposition of the NBTI binding sites from the *E. coli* closed conformation (this study) in green and from the crystal structure of the *S. aureus* DNA-binding/cleavage domain in pink (PDB ID 6QTK) [9]. Residues in the close vicinity of the NBTI are annotated. The first number corresponds to the *E. coli* residue and the second to the *S. aureus* residue. The major difference resides on the I74 in *E. coli* that corresponds to a methionine residue (Met75) in *S aureus.* **b.** Sequence alignment on the NBTI binding region of *E. coli* (Uniprot: P0AES6), *S. Aureus* (Uniprot: P0A0K8) and *N. Gonorrhoeae* (Uniprot: P22118). **c**. Phylogenetic tree generated using the multiple alignment from (b). *N. Gonorrhoeae* and *E. coli* are evolutionary closer than *S. aureus*.

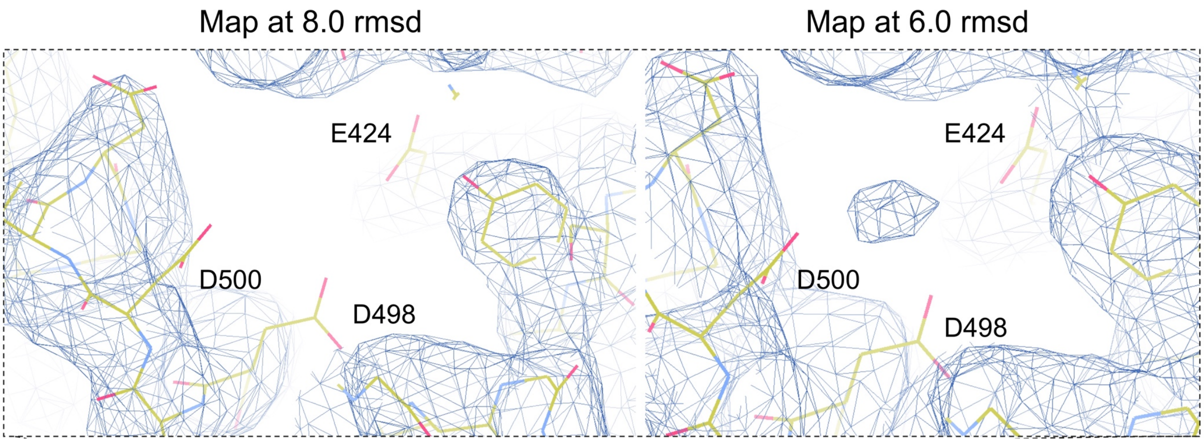

Supplementary Figure 12. Metal-binding site associated with cleavage of DNA by DNA gyrase. EM density of the cleavage-complex structure solved at 4.0 Å resolution at different rsmd steps of contouring showing a density at the site of a Mg^{2+} ion at 6.0 rmsd.

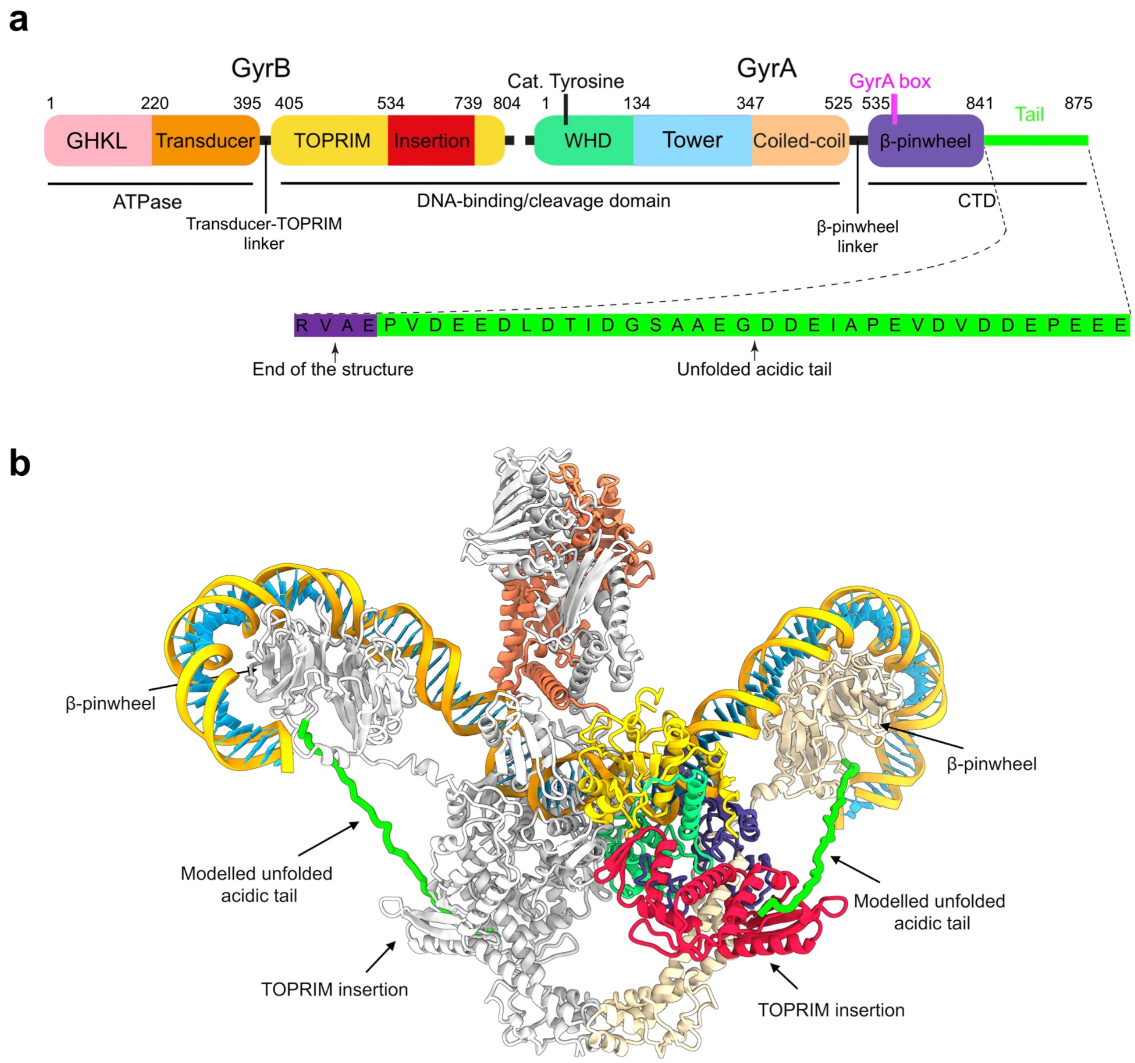

Supplementary Figure 13. CTD β-pinwheel acidic tail modeling. a. Primary domain structure of the *E. coli* DNA gyrase. The β-pinwheel acidic tail is highlighted in green. Sequence of the *E. coli* GyrA β-pinwheel tail is shown in the corresponding colors. **b.** Structure of the *E. coli* DNA gyrase complex with the 130 bp DNA duplex. The unfolded β-pinwheel acidic tails were modelled as coils (green). The distance between the C-terminal end of the β-pinwheel and the GyrB TOPRIM insertion domain is compatible with the 34 residues sequence of the tail, triggering potential contact between the 2 domains.

a

b

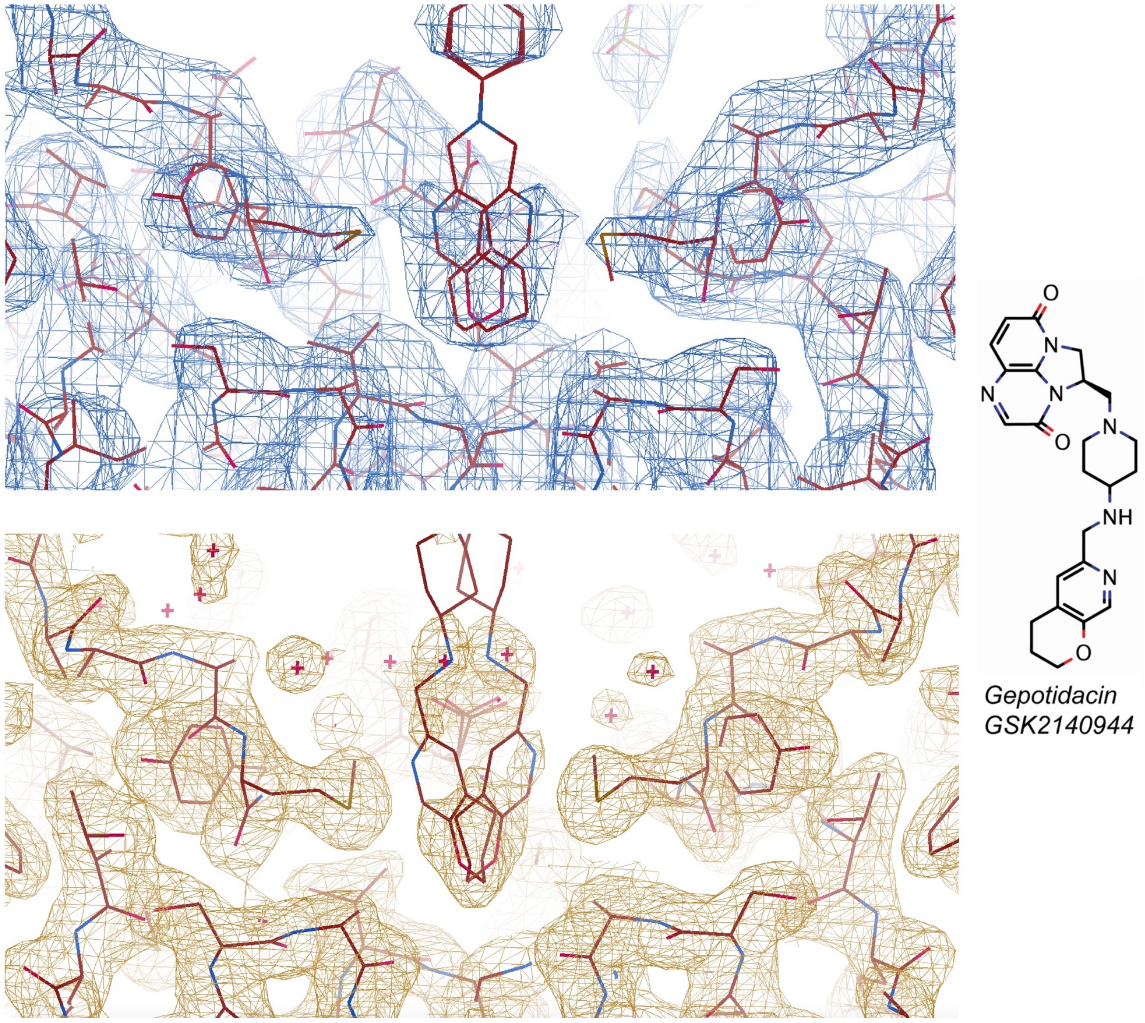

Gepotidacin
GSK2140944

Supplementary Figure 14. Experimental map quality around the NBTI molecule. a. Cryo-EM map at 1.6 rmsd of the 4.0 Å structure of the DNA-binding/cleavage domain in closed state (our study). **b.** Electron density map contoured at 1 σ from the crystal structure of the *S. aureus* DNA-binding/cleavage domain (PDB ID 6QTK) [9]. The same level of details can be obtained in this region when comparing the X-ray and the cryo-EM data.

Supplementary Table 1. Asymmetric oligonucleotides sequences.

92bp-AsymDuplex	GTACCTACGGCTTATGATTCTTCTCGCTTCCGGCGGCATCGG GATGCCCGCGTTGCAGGCCATGCTGTCCAGGCAGGTAGATGA CGACCATC
88bp-AsymDuplex	GATGGTCGTCATCTACCTGCCTGGACAGCATGGCCTGCAACG CGGGCATCCCGATGCCGCCGGAAGCGAGAAGAATCATAAGC CGTAG

Supplementary Table 2. Data collection, processing and refinement statistics.

**Data Collection**	
Microscope	Titan Krios
Voltage (keV)	300
Magnification	130,000
Electron dose (e^{-} Å^{-2})	50
Detector	Gatan K2 Summit (super-resolution mode)
Pixel Size (Å)	0.88 (0.44)
Defocus target (nm)	-500

**Individual data sets**				
Batches	**1**	**2**	**3**	**4**
Micrographs (no.)	3475	2528	2922	3980
Extracted particles (no.)	300,591	278,638	369,008	624,725
Particles after cleaning	116,535	84,192	110,488	168,452

**Merged data sets for refinement**	
Total merged particles	479,667
Particles after cleaning (one round of 2D and 3D ab initio classification)	192,456

**Reconstruction**						
	**Closed Core ΔInsertion**	**Closed Core**	**Pre-opening Core**	**Overall complex**	**ATPase & Core**	**CTD & Core**
EMDB	EMD-4909	EMD-4910	EMD-4912	EMD-4913	EMD-4914	EMD-4915
PDB	6RKS	6RKU	6RKV	6RKW	-	-
Software	Relion2.0.3					
Final particles (no.)	60,548	60,548	53,655	94,633	58,329	45,040
Box size (pixels)	360x360x360					
Symmetry imposed	C2	C2	C2	C1	C1	C1
Map resolution FSC 0.143 (global) (Å)	4.0	4.0	4.6	6.6	5.9	6.3
Applied B-factor for sharpening (Å^2)	-144.9	-79.4	-167.2	-246.5	-234.6	-144.9

**Model refinement**						
Software	Phenix 1.12-2829				-	-
Resolution cut-off (Å)	4.0	4.0	4.6	6.6		
Unit cell (Å)	316.8x316.8x316.8					
Non-hydrogen atoms	12572	15526	15460	30270		
Protein residues	1416	1784	1784	3218		
DNA bases (atoms)	1312	1312	1312	4920		
Ligands (atoms)	66	66	0	128		
Average B-factor	69.03	145.1	187.4	532.8		
R.m.s. deviations						
Bond lengths (Å)	0.004	0.004	0.003	0.003	-	-
Bond angles (°)	0.936	0.904	0.877	0.839		

**Validation**						
Real space correlation coefficient (Global)	0.83	0.84	0.80	0.77	-	-
MolProbity score	1.78	1.80	1.76	1.61		
Clashscore (all atoms)	5.24	5.93	7.23	7.62		
Poor rotamers (%)	0	0	0	0.07		
Ramachandran plot						
Favoured (%)	91.64	92.35	94.67	96.83	-	-
Allowed (%)	8.36	7.65	5.33	3.17		
Outliers (%)	0	0	0	0		

Supplementary Table 3. Missing DNA gyrase sequence elements modeled in this study.

Uniprot ID	Protein name	Domain	PDB ID	Residues range	Comment	Reference
GYRB_ECOLI	GyrB Total of 37 residues added	ATPase	1EI1	2-392		[7]
		Linker		393-401 (9)	manually built	this work
		DNA-binding/cleavage	3NUH	402-448		[6]
				449-461 (13)	manually built	this work
			3NUH	462-475		[6]
				483-488 (6)	manually built	this work
			3NUH	489-655		[6]
				656-658 (3)	manually built	this work
			3NUH	659-765		[6]
				766-784	3NUH manually corrected (Supplementary Fig. 7)	this work
				785-790 (6)	manually built	this work
GYRA_ECOLI	GyrA Total of 49 residues added	DNA-binding/cleavage		8-18 (11)	manually built	this work
			3NUH	19-174		[6]
				175-178 (4)	manually built	this work
			3NUH	179-251		[6]
				252-255 (4)	manually built	this work
			3NUH	256-415		[6]
				416-419 (4)	manually built	this work
			3NUH	420-422		[6]
				427-429 (3)	manually built	this work
			3NUH	430-441		[6]
				442-443+446 (3)	manually built	this work
			3NUH	447-524		[6]
		Linker		525-534 (10)	manually built	this work
		β-pinwheel	1ZI0 (chain B)	535-563		[10]
				564-574 (11)	modeled by Phyre2	this work
			1ZI0 (chain B)	575-841		[10]

Supplementary Table 4. Primer sequences used for the plasmids mutagenesis.

Tth GyrB K284K.fw	CTCACCGCCTTCAGGTCCGCCTACAGC
Tth GyrB K284K.rev	GCTGTAGGCGGACCTGAAGGCGGTGAG
Tth GyrB K284Q.fw	CCTCACCGCCTTCCAGTCCGCCTACAG
Tth GyrB K284Q.rev	CTGTAGGCGGACTGGAAGGCGGTGAGG
Ec GyrB R286K.fw	ACGGGTCATCGCCGCCTTGAAGCCTGCCAGGTG
Ec GyrB R286K.rev	CACCTGGCAGGCTTCAAGGCGGCGATGACCCGT
Ec GyrB R286Q.fw	GGGTCATCGCCGCCTGGAAGCCTGCCAG
Ec GyrB R286Q.rev	CTGGCAGGCTTCCAGGCGGCGATGACCC
Ec GyrB E264A.fw	GGAACGATGGCTTCCAGGCAAACATCTACTGCTTTAC
Ec GyrB E264A.rev	GTAAAGCAGTAGATGTTTGCCTGGAAGCCATCGTTCC

Supplementary References

1. Punjani, A., Rubinstein, J.L., Fleet, D.J. & Brubaker, M.A. cryoSPARC: algorithms for rapid unsupervised cryo-EM structure determination. *Nat Methods* **14**, 290-296 (2017).
2. Zhang, K. Gctf: Real-time CTF determination and correction. *J Struct Biol* **193**, 1-12 (2016).
3. Scheres, S.H. RELION: implementation of a Bayesian approach to cryo-EM structure determination. *J Struct Biol* **180**, 519-30 (2012).
4. Kimanius, D., Forsberg, B.O., Scheres, S.H. & Lindahl, E. Accelerated cryo-EM structure determination with parallelisation using GPUs in RELION-2. *Elife* **5**(2016).
5. Heymann, J.B. & Belnap, D.M. Bsoft: image processing and molecular modeling for electron microscopy. *J Struct Biol* **157**, 3-18 (2007).
6. Schoeffler, A.J., May, A.P. & Berger, J.M. A domain insertion in Escherichia coli GyrB adopts a novel fold that plays a critical role in gyrase function. *Nucleic Acids Res* **38**, 7830-44 (2010).
7. Brino, L. et al. Dimerization of Escherichia coli DNA-gyrase B provides a structural mechanism for activating the ATPase catalytic center. *J Biol Chem* **275**, 9468-75 (2000).
8. Chen, S.F. et al. Structural insights into the gating of DNA passage by the topoisomerase II DNA-gate. *Nat Commun* **9**, 3085 (2018).
9. Gibson, E.G., Bax, B., Chan, P.F. & Osheroff, N. Mechanistic and Structural Basis for the Actions of the Antibacterial Gepotidacin against Staphylococcus aureus Gyrase. *ACS Infect Dis* **5**, 570-581 (2019).
10. Ruthenburg, A.J., Graybosch, D.M., Huetsch, J.C. & Verdine, G.L. A superhelical spiral in the Escherichia coli DNA gyrase A C-terminal domain imparts unidirectional supercoiling bias. *J Biol Chem* **280**, 26177-84 (2005).
