# Supplementary material for: Cryo-EM structure of the complete E. coli DNA gyrase nucleoprotein complex
Source: Nat Commun. 2019 Oct 30;10:4935. doi: 10.1038/s41467-019-12914-y (PMC6821735; doi:10.1038/s41467-019-12914-y)
Supplement: Supplementary file 3 — Description of Additional Supplementary Files [file 41467_2019_12914_MOESM3_ESM.pdf]

**Description of Additional Supplementary Files**

**File name:** Supplementary Movie 1

**Description:** Composite cryo-EM structure of the *E. coli* DNA gyrase nucleoprotein complex and the complete refined atomic model.

**File name:** Supplementary Movie 2

**Description:** Visualization of structural transitions between the *apo* and closed DNA-bound conformation of the gyrase DNA-binding and cleavage domain (1-20 s) and of the closed to pre-opening conformation of the gyrase DNA-binding and cleavage domain (23-46 s).
